# Finger millet RNA-seq reveals differential gene expression associated with tolerance to aluminum toxicity and provides novel genomic resources

**DOI:** 10.3389/fpls.2022.1068383

**Published:** 2022-12-09

**Authors:** Haftom Brhane, Teklehaimanot Haileselassie, Kassahun Tesfaye, Rodomiro Ortiz, Cecilia Hammenhag, Kibrom B. Abreha, Ramesh R. Vetukuri, Mulatu Geleta

**Affiliations:** ^1^ Biology Department, Aksum University, Aksum, Ethiopia; ^2^ Institute of Biotechnology, Addis Ababa University, Addis Ababa, Ethiopia; ^3^ Department of Plant Breeding, Swedish University of Agricultural Sciences, Lomma, Sweden; ^4^ Ethiopian Biotechnology Institute, Ministry of Innovation and Technology, Addis Ababa, Ethiopia

**Keywords:** aluminum toxicity, differentially expressed genes, *eleusine coracana*, RNA-seq, transcriptome

## Abstract

*Eleusine coracana*, finger millet, is a multipurpose crop cultivated in arid and semi-arid regions of Africa and Asia. RNA sequencing (RNA-seq) was used in this study to obtain valuable genomic resources and identify genes differentially expressed between Al-tolerant and Al-susceptible genotypes. Two groups of finger millet genotypes were used: Al-tolerant (215836, 215845, and 229722) and Al-susceptible (212462, 215804 and 238323). The analysis of the RNA-seq data resulted in 198,546 unigenes, 56.5% of which were annotated with significant hits in one or more of the following six databases: NR (48.8%), GO (29.7%), KEGG (45%), PlantTFDB (19.0%), Uniprot (49.2%), and NT (46.2%). It is noteworthy that only 220 unigenes in the NR database had significant hits against finger millet sequences suggesting that finger millet’s genomic resources are scarce. The gene expression analysis revealed that 322 genes were significantly differentially expressed between the Al-tolerant and Al-susceptible genotypes, of which 40.7% were upregulated while 59.3% were downregulated in Al-tolerant genotypes. Among the significant DEGs, 54.7% were annotated in the GO database with the top hits being ATP binding (GO:0005524) and DNA binding (GO:0003677) in the molecular function, DNA integration (GO:0015074) and cell redox homeostasis in the biological process, as well as cellular anatomical entity and intracellular component in the cellular component GO classes. Several of the annotated DEGs were significantly enriched for their corresponding GO terms. The KEGG pathway analysis resulted in 60 DEGs that were annotated with different pathway classes, of which carbohydrate metabolism and signal transduction were the most prominent. The homologs of a number of significant DEGs have been previously reported as being associated with Al or other abiotic stress responses in various crops, including carboxypeptidase SOL1, HMA3, AP2, bZIP, C3H, and WRKY TF genes. A more detailed investigation of these and other DEGs will enable genomic-led breeding for Al tolerance in finger millet. RNA-seq data analysis also yielded 119,073 SNP markers, the majority of which had PIC values above 0.3, indicating that they are highly informative. Additionally, 3,553 single-copy SSR markers were identified, of which trinucleotide SSRs were the most prevalent. These genomic resources contribute substantially to the enrichment of genomic databases for finger millet, and facilitate future research on this crop.

## Introduction

Finger millet (*Eleusine coracana* (L.) Gaertn) is a highly nutritious and multipurpose cereal crop cultivated in arid and semi-arid tropical regions of Africa and Asia (Hilu and De Wet, 1976; Hilu et al., 1979). In Ethiopia, it is commonly grown throughout Tigray, in parts of Oromia (Wellega, IIluababora, and Hararghe), Amhara (Gonder and Gojjam) and the Southern Nations and Nationalities and People’s region (Gamo-Gofa and Hossana) (Admassu et al., 2009; Kinfe et al., 2017). It is a hardy crop capable of providing reasonable grain yield under circumstances where most crops give negligible yield. Finger millet is a staple food crop in drought prone areas of the world and is often considered a component of food security strategies (Kinfe et al., 2017). In Ethiopia, three finger millet types, as per their grain color, are dominant and widely cultivated (black, brown, and white). The black grain as described by farmers, is suited for making local drinks due to having better fermentation quality, as well as its storability and straw quality is preferred (Kinfe et al., 2017). Farmers considered this type tolerant to bird attack which might be due to its high tannin content. On the other hand, the white grain type with low tannin content has been reported to be highly preferred by birds (Tsehaye et al., 2006).

Finger millet is exceptionally rich in nutrients, dietary fiber, and proteins, and is a source of essential amino acids (Nakarani et al., 2021). Consequently, it is classified as a nutraceutical crop, which has a significant contribution to promoting human health. Even though it is a nutraceutical and climate resilient crop, its yield is low. This is due to various factors including less emphasis given to the crop for improvement, and hence shortage of improved cultivars, as well as biotic and abiotic stresses (Kinfe et al., 2017). Soil acidity is among the abiotic factors that significantly limit crop growth and productivity worldwide. It has been estimated that in excess of 50% of the world’s potentially arable lands are acidic (Sade et al., 2016). The western part of Ethiopia are among the top finger millet-producing region, but about 34% of its arable land soils are acidic although the acidity level differs (Abdenna et al., 2007). Hence, developing cultivars tolerant to acidic soils is crucial in order to boost the overall production and productivity of the crop.

In acidic soils, aluminum (Al) toxicity and phosphorus (P) deficiency are the primary factors that directly affect crop yield and productivity by disrupting plant physiology and phenology (Ma et al., 2001; Kochian et al., 2005; Zhou et al., 2022). This abiotic stress is becoming critical due to flooding, removal of crop products from the farm, leaching of nitrogen below the plant root zone and build-up of inorganic matter (Gupta et al., 2013; Bose et al., 2015; Too et al., 2018). Due to Al-toxicity up to 80% grain yield losses have been reported in rice (Kang et al., 2011) and wheat (Valle et al., 2009), however to our knowledge there are no published researches on finger millet in relation with Al-tolerance. Aluminum is phytotoxic to plants because it disrupts or inhibits root growth, increases the rigidity of cell wall and cell membrane, interrupts cell division, induces oxidative stress, and blocks the influx of essential nutrients (Delhaize and Ryan, 1995; Zhang et al., 2014; Eekhout et al., 2017). Plants have different tolerance mechanisms against such abiotic stresses. The most effective mechanisms of Al-tolerance depend on the type and amount of organic acid anions (chelating root exudates, such as citrate, oxalate, and malate) released from root tips, which can chelate and thus neutralize Al-ion (Kochian et al., 2004; Horst et al., 2010). Soluble Al interacts with the cell *via* a receptor protein (R) on the plasma membrane, then activates the transcription of genes that encode proteins involved in the metabolism of organic acids and their transport across the plasma membrane (Ma et al., 2001).

Transcription factors (TFs) have been identified in different crops playing a vital role in activating the expression of Al-tolerant genes to tolerate Al-toxicity (Fang et al., 2020; Sun et al., 2021). Although, plant species have different numbers and types of TF families, the major families of TFs are AP2/EREBP or AP2/ERF, ABI3VP1, ARF, bZIP/HD-ZIP, C2H2, GRAS, MYB/MYC, Zinc fingers, MADS, NAC and WRKY (Gahlaut et al., 2016). Of these TFs, bZIP, AP2, MYB/MYC, NAC, and WRKY have been associated with developmental processes and are also involved in biotic and abiotic stresses tolerance including drought, Al-tolerance, salt tolerance, and disease (Zhu et al., 2013; Huang et al., 2015). The MATE family TFs also have a more general role in plant adaptation to low pH soils (Magalhaes et al., 2007).

Transcriptome sequencing has opened a new way for the development of DNA markers such as simple sequence repeat (SSR) or microsatellites, and single nucleotide polymorphism (SNP). Microsatellites are co-dominant DNA markers of short tandem repeats consisting of 2–6 bp nucleotides that are extensively present across whole genomes, including within genes (Tsehay et al., 2020; Gebeyehu et al., 2022). They are major components of a genome and they play significant roles in the evolution and adaptation of plant species. Transcriptome sequencing generates a large amount of sequence data, which can be used as a source of SSRs to enrich genomic resources of crops for various applications. SNPs are the most recent and popular co-dominant markers extensively used for diversity analysis, genome-wide association studies, and genetic linkage mapping in various crops including finger millet (Enyew et al., 2021; Brhane et al., 2022). By utilizing high-throughput sequencing technology, RNA-seq provides a platform for the development and utilization of SSRs and SNPs for species lacking genomic information at a fast, accurate, and affordable scale. Using the Illumina platforms for transcriptome-based DNA marker development, sequence variations (e.g. SNPs and SSRs) have been discovered in several crops, such as sugarcane (*Saccharum officinarum* L.), sweet potato and noug (*Guizotia abyssinica*) (Xu et al., 2018; Kong et al., 2020; Tsehay et al., 2020). DNA markers identified from transcriptomic sequences are more efficient, cost-effective, flexible, and more informative than markers derived from genomic sequence data for gene-based interpretation and detecting functional variation (Zheng et al., 2013).

RNA-sequencing (RNA-seq) is an efficient and less expensive next-generation sequencing (NGS) method that has broad applications, including the identification of differentially expressed genes and determining pathways in crops grown under different stress (Khatiwada et al., 1996; Mattiello et al., 2010; Xu et al., 2017; Gharaghanipor et al., 2022). There has been no published transcriptome-based research on Al-tolerance in finger millet to the best of our knowledge. Hence, the aim of this research was to identify differentially expressed genes (DEGs) and determine their functional annotation through nucleotide and sequence databases from three Al-tolerant and three Al-susceptible finger millet genotypes. This research also aimed at determining the frequency and distribution of simple sequence repeat (SSR) markers in the finger millet’s transcriptome sequences. Furthermore, this study aimed at developing transcriptome-based SNPs using Al-tolerant and Al-susceptible finger millet genotypes, which serve as new genomic resources for various applications, including marker-aided breeding of finger millet in acidic soils.

## Material and methods

### Plant material and their growth conditions

In this study, leaves collected from six finger millet accessions were used for RNA-seq-based transcriptome analysis. Originally, the accessions were collected from locations representing different agro-ecologies in Ethiopia. These are accessions 215836 (from Gojjam), 215845 (from Bahir dar), 229722 (from Metekel), 212462 (from Hararge), 215804 (from Wellega), and 238323 (from Tigray). They were selected for sampling their representative genotypes based on the results of a preliminary hydroponic experiment, which was carried out as described in (Brhane et al., 2022). The results showed that the first three accessions (215836, 215845, and 229722) could be regarded as Al-tolerant whereas, the other three (212462, 215804, and 238323) are Al-susceptible.

For this study, the germination of the seeds sampled from the selected accessions followed by the exposure of their seedlings to Al-toxicity was carried out as described in Brhane et al. (2022). After germination, the seedlings were transferred to a nutrient solution containing 500 μM KNO_3_, 500 μM CaCl_2_, 500 μM NH_4_NO_3_, 150 μM MgSO_4_.7H_2_O, 10 μM KH_2_PO_4_, 2 μM FeCl_3_, and 100 μM Al_2_ (SO_4_)_3_.18H_2_O, which was prepared according to Zhou et al. (2013). The pH of the nutrient solution was adjusted to 4.3 (using 1M HCl or NaOH) and renewed every day to maintain the pH and Al-concentration relatively constant. After 10 days of treatment, the relative root growth of the seedlings was estimated following the methods of Mendes et al. (1984).

The three genotypes sampled from accessions: 215836, 215845, and 229722 were confirmed to be Al- tolerant, and hereafter will be referred to as AT1, AT2, and AT3, respectively. Whereas, the other three genotypes sampled from accessions 212462, 215804, and 238323 were confirmed to be Al-susceptible, and hereafter will be referred to as AS1, AS2, and AS3, respectively. The seedlings of each Al-tolerant and Al-susceptible genotypes were transferred to separately labeled 5 L soil-filled plastic pots for further growth. The seedlings were allowed to grow for about four weeks in a greenhouse adjusted to a temperature of 18°C and a humidity level of 65% at the Swedish University of Agricultural Sciences (SLU, Alnarp, Sweden).

### Sampling and RNA extraction

**Figure 1:**
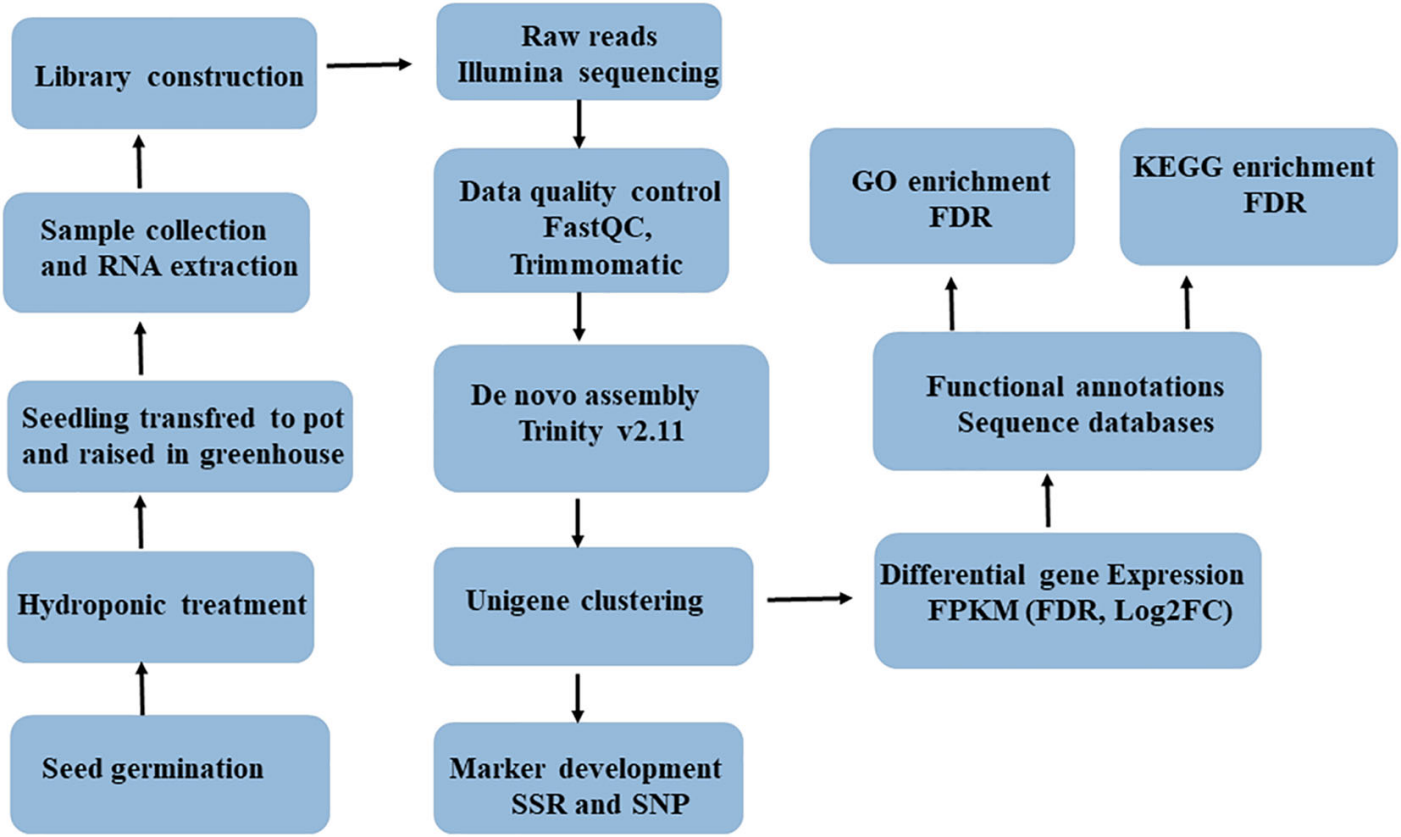
Complete workflow of RNA-seq data acquisition and analysis involving six finger millet genotypes (AT1, AT2, AT3, AS1, AS2 and AS3).

The leaf tissue of five-weeks-old seedlings of each genotypes was snap-frozen in liquid nitrogen and then kept at −80°C until RNA was extracted. A total RNA sample was extracted from ca100 mg leaf tissue of each genotype using the RNeasy Plant Mini Kit (#74904, QIAGEN, Valencia, CA), and subsequently treated with DNase using the Ambion Turbo DNA-Free Kit (#AM1907, Thermo Fisher Scientific, United States). An Agilent Bioanalyzer 2100 system (Agilent Technologies, United States), a NanoDrop ND-1000 spectrophotometer (Saveen Werner, Sweden), and agarose gel electrophoresis were used to evaluate the quality and concentration of extracted RNA. The high-quality RNA samples were then shipped to CD Genomics (New York, USA) on dry ice for RNA sequencing. Upon arrival at the CD Genomics, the samples were checked for degradation and contamination using agarose gels (1%), purity was checked using the NanoPhotometer spectrophotometer (IMPLEN, CA, USA), concentration was measured using the Qubit RNA Assay Kit in Qubit 2.0 Flurometer (Life Technologies, CA, USA), and integrity was assessed using the RNA Nano 6000 Assay Kit of the Agilent Bioanalyzer 2100 system (Agilent Technologies, CA, USA). The workflow for RNASeq data acquisation and analysis is provided in **Figure 1**.

### Library construction and RNA sequencing

To construct the sequencing library, 1.5 mg of RNA was utilized per sample, and index codes were added to assign sequences to each sample using the NEBNextR UltraTM RNA Library Prep Kit for Illumina (NEB, United States) according to the manufacturer’s instructions. A Beckman Coulter AMPure XP system (Beverly, United States) was used to purify the library fragments in order to facilitate preferential selection of cDNA fragments with 150-200 bp size. The amplified products were purified using the AMPure XP system following adaptor ligation and polymerase chain reaction (PCR), and then quality of the purified library was assessed with the Agilent Bioanalyzer 2100. The index-coded samples were then clustered using a cBot Cluster Generation System using the TruSeq PE Cluster Kit v3-cBot-HS (Illumina) according to the manufacturer’s instructions. The clusters were subsequently paired-end sequenced using Illumina HiSeq™ 2500 v2 platform (Illumina Inc., United States).

### Processing and assembly of transcriptome data

The raw data generated from the high throughput sequencing method were transformed into sequence reads by base calling. The sequencing quality was checked through a series of filtering techniques to obtain high-quality sequence reads using FastQC v.0.10.1 (Andrew, 2010). The sequencing quality scores of the raw reads were determined through the Phred score approach. Adaptors, short reads (less than 60 bp), duplicate, and low quality reads was trimmed using Trimmomatic v. 0.3 (Bolger et al., 2014). Only reads with a read-mapping depth of above 10 were selected to generate unique transcript sequences. Since no chromosome-level reference genome is available for finger millet, the high-quality reads were assembled into transcripts and spliced to obtain a *de novo* reference transcriptome using Trinity v. 2.1.1 with default settings (Grabherr et al., 2011). A length distribution analysis was performed for the transcripts of each gene to determine the longest spliced transcript, which is referred to as a unigene. These final unique transcript sequences representing different genes (a unigene set) were used for downstream analysis. The unigene set has been deposited at DDBJ/EMBL/GenBank, as a Transcriptome Shotgun Assembly project under the accession GJZB00000000. The RNA-seq quality-trimmed raw reads were also deposited in Sequence Reads Archive (SRA), BioProject PRJNA839867.

### Functional annotation and pathway analysis

Functional annotation was employed to categorize the function of the assembled unigenes using: Gene Ontology (GO; http://www.geneontology.org/; (Consortium, G.O 2019), Nucleotide (NT; https://www.ncbi.nlm.nih.gov/), Non-Redundant Protein (NR; https://www.ncbi.nlm.nih.gov/; (Bleasby and Wootton, 1990), Kyoto Encyclopedia of Genes and Genomes (KEGG; http://www.genome.jp/kegg/; (Kanehisa and Goto, 2000), Universal Protein (UniProt, http://www.ebi.ac.uk/uniprot/; (Consortium, U 2015), and plant transcription factor database (PlantTFDB v.3.0, http://planttfdb.gao-lab.org; (Tian et al., 2020). Furthermore, analyses were conducted to determine the databases used to annotate each unigene and the species that contributed most to the annotations.

### Hierarchal clustering and principal component analysis

The unweighted pair group method with arithmetic mean (UPGMA) cluster analysis of the expressed genes from the finger millet genotypes was carried out using the UPGMA algorithm implemented in the “vegan” package in R (Oksanen et al., 2007) using Euclidean pairwise distances. Principal component analysis (PCA) was also performed to determine the overall transcriptome based relationship of the genotypes using the “adegenet” package in R.

### Gene expression quantification and differential expression analysis

To quantify the abundance of the transcript in the two (Al-tolerant and Al-susceptible) groups, the sequenced pair-end reads were mapped onto the assembled transcriptome, and the read count for each gene was obtained from the mapping results. RNA Sequencing by Expectation Maximization package (RSEM v.1.2.08; (Li and Dewey, 2011) was used to estimate the expression level of each transcript. DEGseq R package was used to perform differential expression analysis of the Al-tolerant and Al-susceptible genotypes. The abundance level of each gene was estimated by calculating the fragment per kilobase pair per million reads (FPKM), and those transcripts with FPKM values equal to or larger than 0.5 were considered expressed. Genes exhibiting adjusted *p*-values below 0.01 and log2 fold change (log2FC) above 2 were classified as significantly differentially expressed.


$$FPKM=\frac{10^{9}\times \;Counts\;of\;mapped\;fragments}{Total\;mapped\;reads\times \;Exonic\;length\;\left(Kb\right)}$$

The differentially expressed genes were visualized using a volcano plot, which was constructed by plotting the FDR (-log10) on the y-axis, and the expression fold change between the two experimental groups on the x-axis. The regions of interest in the volcano plot are those found towards the top (high statistical significance) and at the extreme left or right (strongly downregulated and upregulated, respectively). Furthermore, two-way hierarchal cluster analysis of the genes and genotypes was performed using the R software package pheatmap v.1.0.8 (Kolde and Kolde, 2015). Likewise, functional annotation and pathway analyses of the significantly differentially expressed genes were carried out using major genomic databases.

GO functional enrichment analysis of DEGs was carried out using topGO. To identify the transcription factor genes among the DEGs, all DEGs were searched against the plant transcription factors database [PlantTFDB v.3.0; (Tian et al., 2020)] with an *e*-value cutoff = 10^-10^, minimum identity = 40, and minimum query coverage = 50%.

### 3D protein structure prediction of significant DEGs

The European Molecular Biology Open Software Suite (EMBOSS) packages “getorf” and “transeq” were used to identify the exact start and stop points of translation and to translate cDNA sequences in to amino acid sequences, respectively (Rice et al., 2000). 3D protein structures of the translated sequences of upregulated and down regulated DEGs were predicted using SWISS-MODEL (SWISS-MODEL (expasy.org)).

### SSR and SNP analysis

The unigenes were scanned to find simple sequence repeats using microsatellite identification tool [MISA v.2.1; http://pgrc.ipk-gatersleben.de/misa; (Beier et al., 2017)]. The SSR search parameters were set to a minimum of 10 for mononucleotide repeats, six for dinucleotide repeats, and five for tri, tetra, penta, and hexanucleotide repeats. Although mononucleotide repeats are not widely used for various SSR applications due to their extremely high abundance, they were analyzed in this study in order to broaden the comparison of the finger millet genome with that of other crops.

The analysis of SNPs within the transcriptome was carried out using the unigenes as reference. The sequence reads of each sample were aligned to a *de novo* assembled reference transcriptome using Burrows-Wheeler Alignment tool [BWA v. 0.7.17; (Li and Durbin, 2009)]. Unmapped, non-unique sequences and duplicate reads generated during PCR were removed using SAMtools v. 1.4 (Li et al., 2009) software and Picard package v. 1.112. Thereafter, clean reads were converted to BAM files and the BAM alignment results of each sample were merged for SNP calling using mutation detection software SAMtools v. 1.4. This was followed by base-quality score calibration and SNP calling using the Haplotype Caller module of the Genome Analysis Toolkit [GATK, v. 3.5; (Mckenna et al., 2010)]. The genotype of each sample at each locus was determined based on standard filtering parameters and variant quality score calibration according to GATK’s Best Practice recommendations (Depristo et al., 2011; Van Der Auwera and O’connor, 2020). Using BCFtools, the SNP loci shared among all samples were filtered by merging the VCF files of the samples (Danecek et al., 2021). Minor allele frequency (MAF) and polymorphism information content (PIC) of each locus were also estimated.

## Results

### Sequencing and transcriptome *de novo* assembly

**Figure 2:**
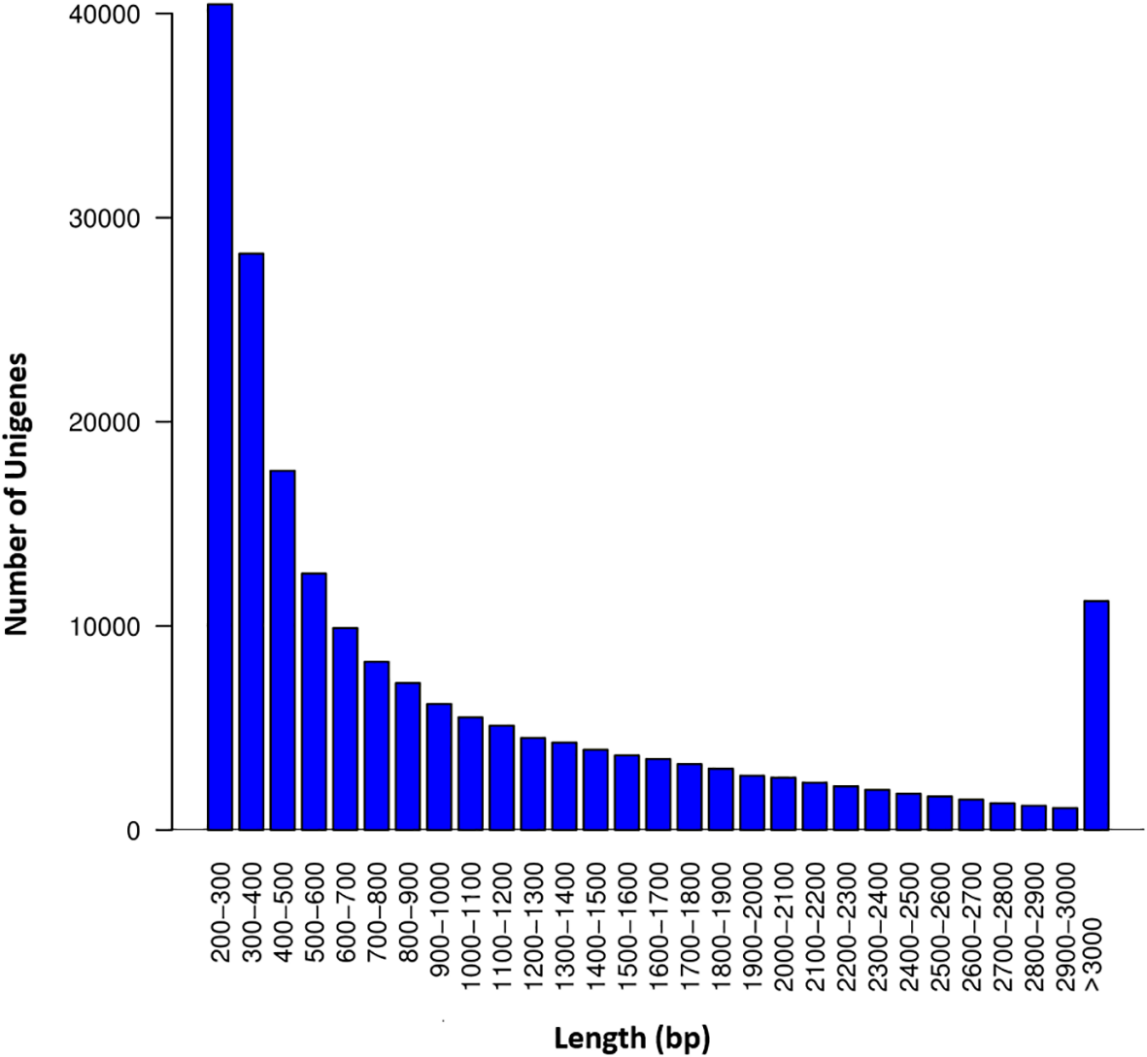
Sequence length distribution of the unigenes. The X-axis represents the length of the unigenes in ranges whereas the Y-axis represents the number of unigenes in each range.

The RNA-seq generated a total of 268,037,778 raw reads with an average of 44.7 million reads per genotype (**Table 1**). An assessment of the data quality revealed that the average GC-content and Phred score of the raw reads met the criteria (GC > 48%, Q30 > 90%), confirming the transcriptome dataset is suitable for further analysis (**Table 1**). After removing adapter sequences, ambiguous and low-quality reads, the high-quality clean reads (Phred score ≥ 30) were spliced using Trinity transcriptome splicing software and were assembled in to 257,291 transcripts corresponding to 198,546 unigenes (**Table 2**). The mean contig length and N50 of the transcripts were 1094.9 bp and 1854 bp, respectively, with 37.5% of the transcripts being large contigs (>1000 bp). Whereas the mean contig length and N50 of the unigenes were 1023.7 bp and 1738 bp, respectively, with 34.3% of the unigenes being large contigs (>1000 bp; **Table 2**). The majority (65%) of the unigenes were 200 to 1000 bp while 35% of them were over 1000 bp in size (**Figure 2**).

**Table 1:** Summary statistics of the raw reads of finger millet transcriptome library: number of reads, GC-content (%), and %age of reads with Phred score ≥30 for each of the six samples, and their mean values.

	Genotypes						
	AT1	AT2	AT3	AS1	AS2	AS3	Mean
Number of reads	45.6×10^6^	49.2×10^6^	41.5×10^6^	42.9×10^6^	45.5×10^6^	43.3×10^6^	44.7×10^6^
GC-content (%)	54.2	54.1	54.3	54.5	54.1	54.2	54.2
Phred score ≥ 30 (%)	94.5	94.1	93.9	94.1	93.5	93.9	94.0

**Table 2:** Summary statistics of the assembled finger millet transcripts and unigenes: total number, number of contigs above 1 kb, maximum contig length, mean contig length, N50, and total length.

	Total Number	Number of contigs above 1 kb	Max contig length (bp)	Mean contig length (bp)	N50 (bp)	Total length (Mb)
Transcripts	257,291	96,463	16,441	1,094.94	1,854	281.7
Unigenes	198,546	68,160	16,441	1,023.69	1,738	203.2

bp, base pairs; kb, kilo base pairs; Mb, mega base pairs.

### Functional annotation and classification of the unigenes

The 198,546 unigenes were BLAST searched in six major databases to determine their potential biological functions. Of these unigenes, 112,243 (56.5%) had significant hits (E-value cutoff = 1e^-5^) at least in one of the databases: NR (96,898; 48.8%), GO (58,992; 29.7%), KEGG (89,253; 45%), PlantTFDB (37,828; 19.0%), Uniprot (97,660, 49.2%) and NT (91,682, 46.2%) (**Table 3**).

**Table 3:** The number of unigenes annotated in each of the six major databases.

Total number of unigenes	Number of unigenes annotated in						Total number of annotated unigenes	%age of annotated unigenes
	NR	GO	KEGG	PlantTFDB	UniProt	NT		
198,546	96,898	58,992	89,253	37,828	97,660	91,682	112,243	56.5

Databases: NR, Non-redundant Protein; GO, Gene Ontology; KEGG, Kyoto Encyclopedia of Genes and Genomes; Uniprot, Universal Proteins; and NT, nucleotide.

**Figure 3:**
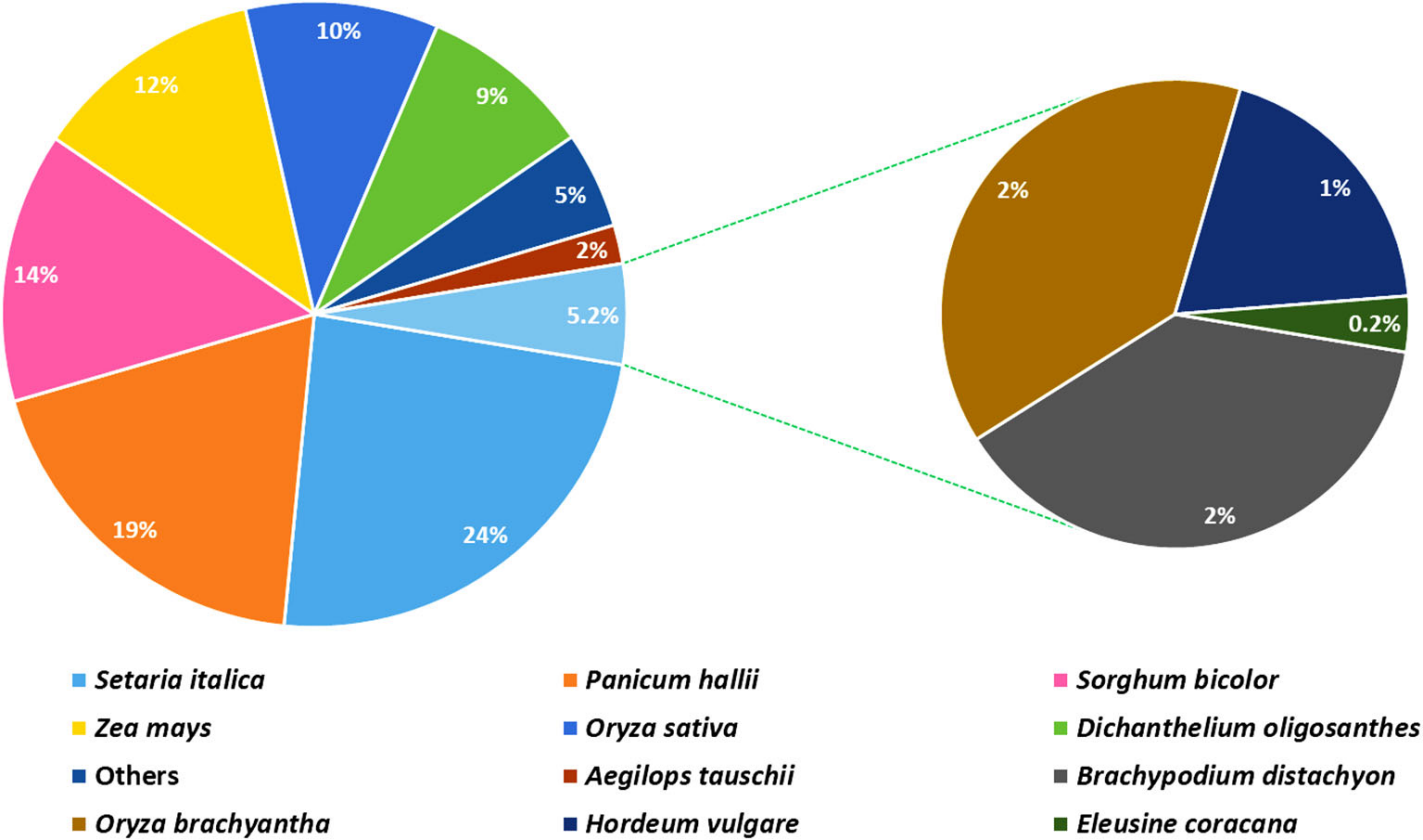
A pie chart depicting the distribution of plant species according to the top BLAST hits against the NCBI Non-redundant Protein database (NR).

The analysis based on the non-redundant protein database revealed that more than 97% of the annotated unigenes had homology to protein sequences of the Poaceae family, such as foxtail millet (*Setaria italic;* 23.7%), perennial Hall’s panicgrass (*Panicum hallii;* 18.8%), sorghum (*Sorghum bicolor;* 13.6%), maize (*Zea mays;* 11.7%), rice (*Oryza sativa;* 9.5%), and perennial Heller’s rosette grass (*Dichanthelium oligosanthes;* 9.4%) (**Figure 3**). Among the unigenes, only 220 (0.2%) and 68 (0.06%) shared significant similarities with sequences of finger millet and its closely related Indian goose grass (*Eleusine indica)*, respectively, available in the genomic databases.

**Figure 4:**
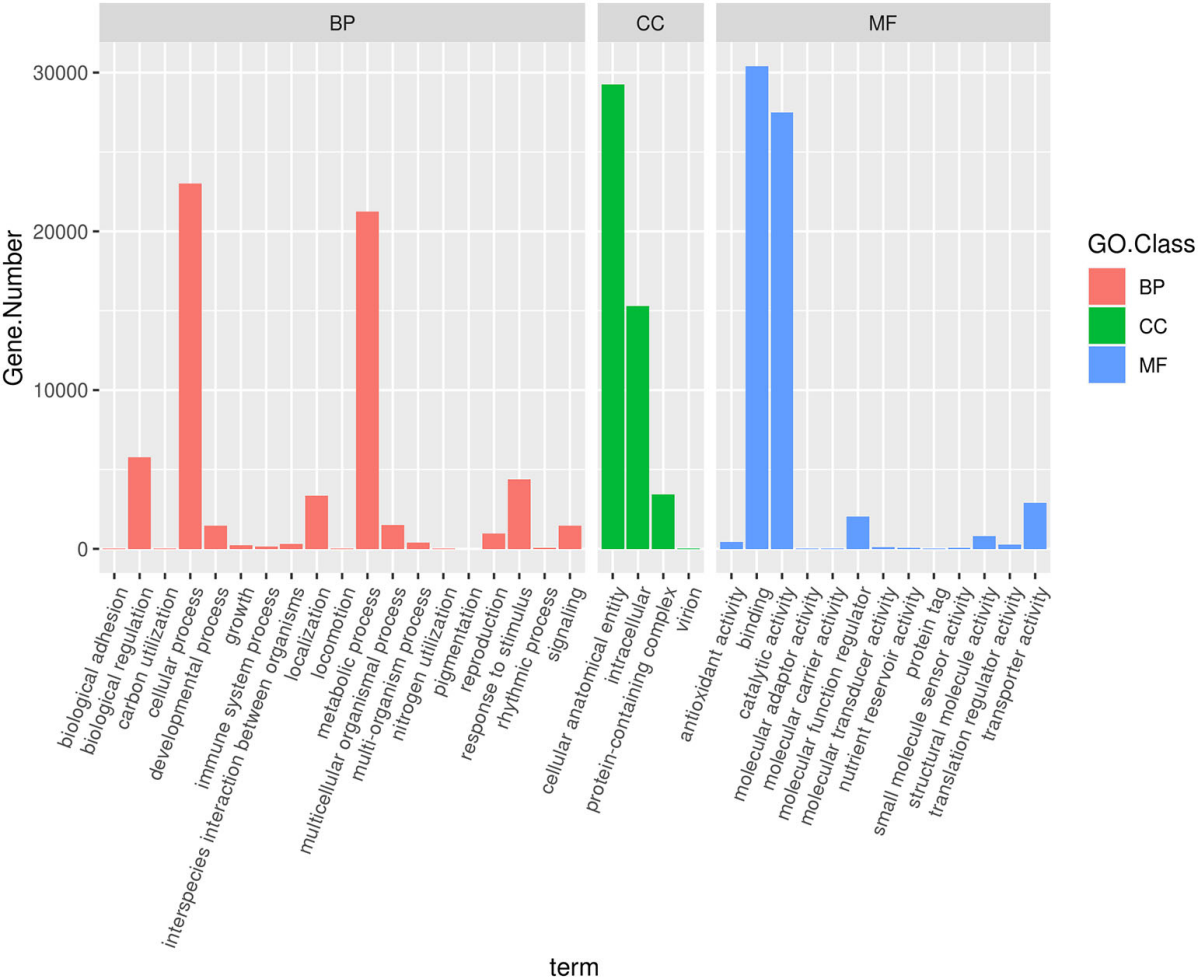
Gene ontology (GO) annotation of finger millet unigenes to biological process (BP), cellular component (CC), and Molecular function (MF) functional categories. X-axis indicates GO terms and Y-axis indicates number of unigenes at each terms.

Of the total 198,546 unigenes, 58,992 (29.7%) were annotated with at least one GO term in the GO database (E-value cutoff = 1e^-5^). The number of unigenes that were annotated under biological process (BP), molecular function (MF), and cellular component (CC) were 65,230, 62,920, and 59,280, respectively (**Figure 4**). Among the 19 sub-functional categories (GO terms) under biological process, the top four that represented a large number of unigenes were cellular process, metabolic process, biological regulation, and response to stimulus. The unigenes were also annotated with cellular anatomical entity, intracellular component, protein-containing complex and virion GO terms in the cellular component (CC) GO class. In the case of the molecular function (MF) category, 13 terms were identified, with the majority of the unigenes annotated with binding, catalytic activity, transporter activity, and molecular function regulator (**Figure 4**).

**Figure 5:**
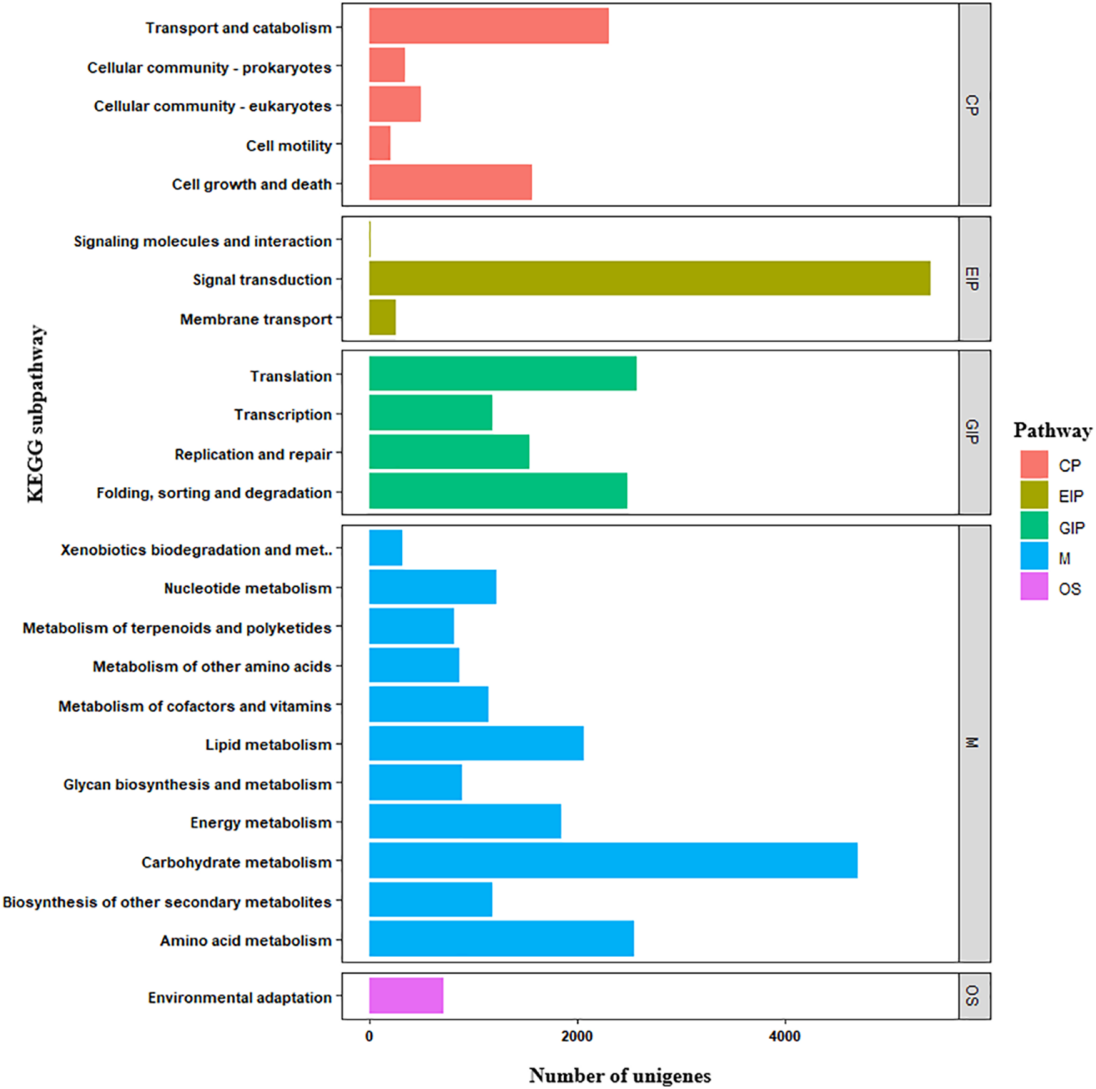
Kyoto Encyclopedia of Genes and Genomes (KEGG) annotation of finger millet unigenes in to different pathway categories. X-axis indicates number of unigenes and Y-axis indicates KEGG sub-pathways.

The unigenes were also annotated separately in the KEGG databases to determine their putative functions based on their sequence homology with annotated genes. Among the 89,253 unigenes with significant BLAST hits (e-value cutoff = 1e^-5^) in the KEGG database, 36,696 (41.1%) unigenes were annotated under 201 pathways. These were classified under five major KEGG categories: cellular processes, environmental information processing, genetic information processing, metabolism and organismal systems (**Figure 5**). Most of the unigenes were annotated with the metabolism pathway. The majority of the unigenes were predicted to be involved in either signal transduction (5401), carbohydrate metabolism (4699), translation (2570), folding, sorting and degradation (2486), transport and catabolism (2302), or lipid metabolism (2067) pathways (**Figure 5**).

The BLAST search of the unigenes in the transcription factor database (http://planttfdb.gao-lab.org/) resulted in significant hits for 37,828 unigenes across 60 different TF family proteins. The top three most frequent transcription factor protein families were bHLH, MYB-related, and NAC, which corresponded to 3580 (9.46%), 2747 (7.26%), and 2588 (6.84%) unigenes, respectively. TFs from 160 plant species were significant matches for the unigenes, with apple (*Malus domestica*), Manila grass (*Zoysia matrella*), and tef (*Eragrostis tef*) being the top three (**Supplementary Figure S1**).

### Gene expression based cluster and principal component analyses

**Figure 6:**
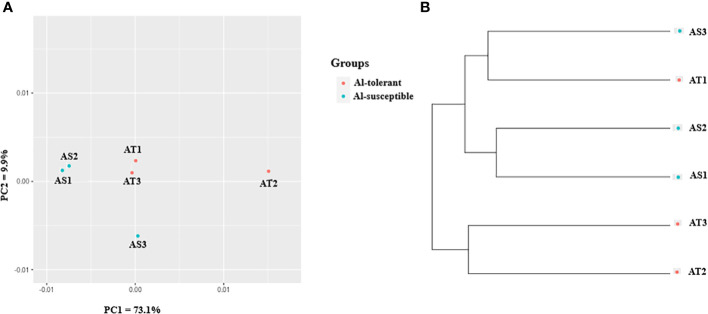
Gene expression-based **(A)** PCA scatter plot, and **(B)** UPGMA cluster depicting the relationship between the finger millet genotypes.

In the context of gene expression, unigenes will be referred to as genes as they represent genes. To visualize the relationship between genotypes in terms of their gene expression, principal component analysis (PCA) was also used (**Figure 6A**). The analysis revealed that the first and second principal components (PC1 and PC2) together explained 83% of the variance in gene expression, with PC1 accounting for most of the variance (73.1%). As with the cluster analysis, AS1 and AS2 were closely grouped together, but separated from the other genotypes along the PC1, while AS3 was separated from the other genotypes along the PC2 (**Figure 6A**). UPGMA clustering of the six genotypes based on gene expression data revealed that they form two clusters, with two of the three Al-tolerant genotypes (AT2 and AT3) being separated from the other four genotypes (**Figure 6B**). Two of the three Al-susceptible genotypes (AS1 and AS2), were the most closely related in terms of their gene expression profiles.

### Differentially expressed genes and their clustering

**Figure 7:**
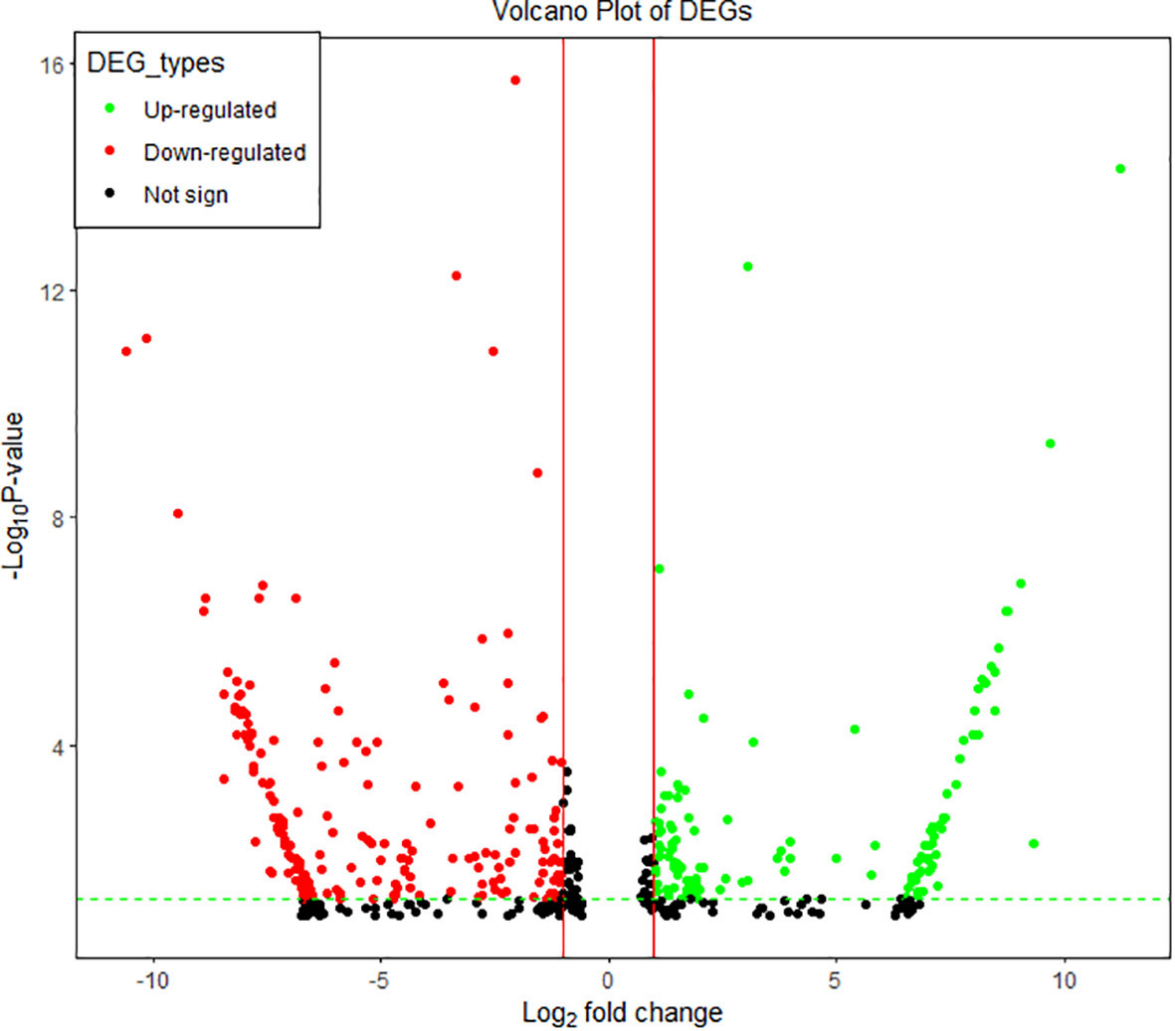
Volcano plots of significantly differentially expressed genes between the two groups of finger millet genotypes. Each dot represents a gene. Dots in green represent genes that were up-regulated in the Al-tolerant group, dots in red represent genes that were down-regulated in the Al-tolerant group, and dots in black represent genes that were not significantly differentially expressed (Not sig).

Comparing Al-tolerant group to the Al-susceptible group, 162,974 (82.1%) genes showed either an up-regulation (80,743 genes) or a down-regulation (82,231 genes) trend. The further filtering of the 162,974 genes based on their adjusted *p*-value and fold-changes resulted in 495 unigenes that were differentially expressed (adjusted *p*-value< 0.1; Log_2_FC< -0.5 or > 0.5) among the two groups (**Supplementary Table S1**). Of the 495 genes, 322 exhibited a fold-change of above two (Log_2_FC< -1 or > 1) with corresponding adjusted *p*-values below 0.05, and hence they are regarded as significantly differentially expressed. Among the 322 unigenes, 131 (40.7%) were significantly upregulated while 191 (59.3%) were significantly downregulated in the Al-tolerant group in comparison with the Al-susceptible group (**Figure 7**; **Supplementary Table S1**).

**Figure 8:**
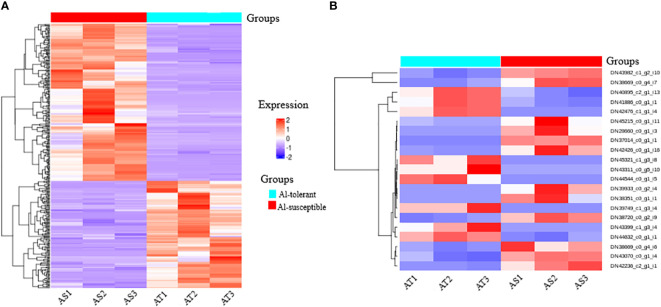
A heatmap map of two-way cluster analysis of **(A)** significantly differentially expressed genes among Al-tolerant and Al-susceptible finger millet genotypes and **(B)** selected unigenes that have association with Al-tolerance. Red shows higher gene expression, while blue shows lower gene expression. Genes are in row forming eight clusters (represented by different colors in a row just above the heatmap rows) based on the similarity in their expression patterns, whereas columns represent the two groups of genotypes and respective genotype.

Hierarchical cluster analysis of the significantly differentially expressed genes revealed eight clusters (**Figure 8A**). The first four clusters represent the downregulated genes and the last four clusters represent the upregulated genes in the Al-tolerant group. It is interesting to note that the three Al-tolerant genotypes showed generally more similar gene expression than the other three Al-susceptible genotypes. The similarity in expression between the three Al-tolerant genotypes is more evident in downregulated genes than in the upregulated genes (**Figure 8A**). For the Al-susceptible group, the three genotypes displayed more similar expressions for genes that were upregulated in the Al-tolerant group.

Moreover, a total of 21 candidate unigenes that might have association with Al-tolerance were selected. The selected unigenes encodes important TFs (WRKY, ERF, bZIP, bHLH, AP2, and C3H), DNA and ATP binding activity, integral components of membrane, Acety-CoA carboxylase activity, signaling pathway, glyoxylate and dicarboxylate metabolism, catalyitic activity, starch and sucrose metabolism (**Figure 8B**). Among the selected unigenes, 9 of them were upregulated in Al-tolerant genotypes whereas, 12 of them were downregulated in Al-tolerant genotypes.

### Annotation of differentially expressed genes

**Figure 9:**
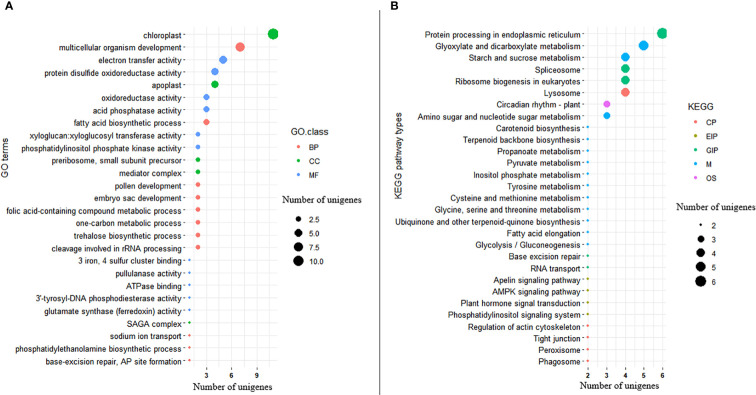
Bubble plot showing **(A)** distribution of GO functional annotation and **(B)** KEGG pathway classification of significantly differentially expressed genes. The Y-axis in GO annotation is the name of the GO terms, and the X-axis is the number of unigenes annotated to each GO term. Similarly, the Y-axis in KEGG pathway classification is the name of the KEGG metabolic pathway, and the X-axis is the number of unigenes annotated to each pathway.

Among the significant DEGs, 176 (54.7%) were annotated with one or more GO terms in the gene ontology database. Among the annotated significant DEGs, 83 of them were annotated to GO terms in MF, 83 of them with GO terms in BP, and 36 of them with GO terms in CC (**Figure 9A**; **Supplementary Table S2**). The top three GO terms annotating the significant DEGs in the MF GO class were ATP binding (GO:0005524; 20 genes), DNA binding (GO:0003677; 14 genes), and nucleic acid binding (GO:0003676; 14 genes). In the BP GO class, DNA integration (GO:0015074; 6 genes) and cell redox homeostasis (GO:0045454; 5 genes) were the top two terms associated with the significant DEGs. The top two GO terms associated with the significant DEGs in the CC class were integral component of membrane (GO:0016021; 42 genes), and nucleus (GO:0005634; 10 genes) (**Supplementary Table S2**). The DEGs annotated with 19 of the 83 Go terms in the MF class were significantly enriched for their corresponding GO terms according to the GO enrichment analysis. The DEGs annotated with 32 of the 83 GO terms in the BP class, as well as with five of the 36 GO terms in the CC class, were also significantly enriched (**Figure 9A**; **Supplementary Table S2**). Protein processing (GO:0016485), metal ion transport (GO:00300001), defense response to bacterium (GO:0042742), regulation of meristem development (GO:0048509), and seedling development (GO:0090351) were among the enriched GOterms indicating their potential role in Al-tolerance (**Supplementary Table S2**).

KEGG pathway analysis was also conducted on the 322 significant DEGs, revealing that 60 DEGs (18.6%) were annotated with 71 pathways belonging to 21 pathway classes (**Supplementary Table S3**). The expression of 30 of these genes was upregulated while that of the remaining 30 genes was downregulated. Among the pathway classes associated with the significant DEGS, metabolism_carbohydrate metabolism (comprising 11 pathways) and environmental information processing_signal transduction (comprising 9 pathways) were the most prominent (**Figure 9B**; **Supplementary Table 3**). The top two pathways associated with the significant DEGs in the metabolism carbohydrate metabolism were glyoxylate and dicarboxylate metabolism (ko00630; 4 genes) and starch and sucrose metabolism (ko00500; 3 genes). As part of the environmental information processing signal transduction pathway class, the phosphatidylinositol signaling system (ko04070), plant hormone signaling system (ko04075), AMPK signaling pathway (ko04152), and apelin signaling pathway (ko04371) were associated with two significant DEGs each. There were five significant DEGs that differentiated Al-tolerant and Al-susceptible groups in the endoplasmic reticulum pathway (belonging to the genetic information processing folding, sorting and degradation pathway class) (**Figure 9B**; **Supplementary Table S3**).

**Figure 10:**
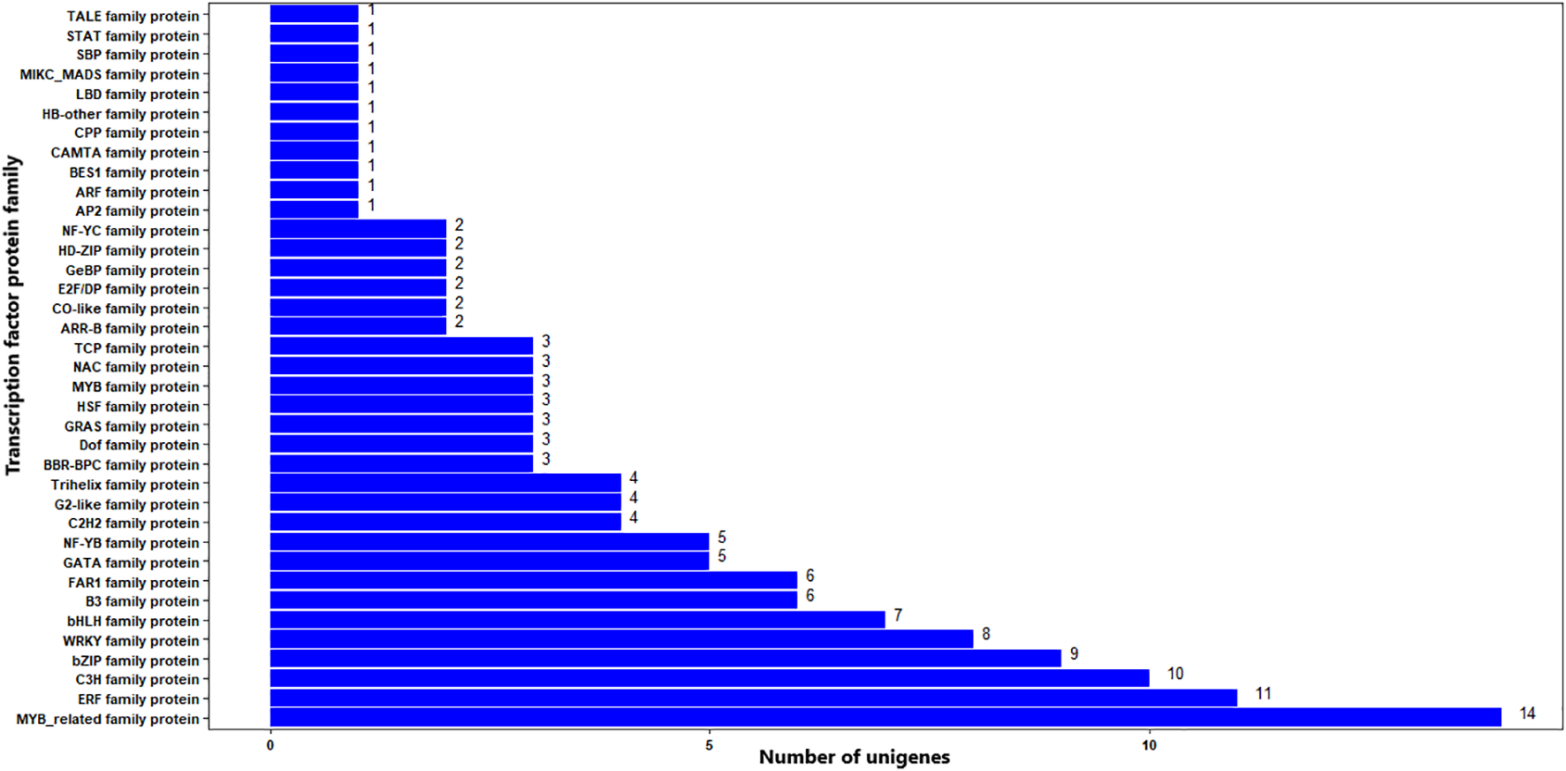
TF distribution of significantly differentially expressed genes. The Y-axis is the name of the TF family proteins, and the X-axis is the number of unigenes annotated to each TF family proteins.

The BLAST search of the 322 significant DEGs in the TF database resulted in that 137 (42.5%) DEGs had significant hits (E-value cutoff = 1e^-5^) against functionally annotated TF genes (**Supplementary Table S4**). The DEGs were associated with 37 TF protein families. The top three most frequent protein families were MYB-related, ERF, and C3H, being associated with 14, 11, and 10 significant DEGs, respectively. Other TF protein families associated with the significant DEGs include bZIP, WRKY, and bHLH with nine, eight, and seven DEGs, respectively (**Figure 10**; **Supplementary Table S4**). TFs from 61 plant species were significant matches for the significant DEGs, with wild or woodland strawberry (*Fragaria vesca*), Manila grass, and apple, being the top three with eight, seven, and seven matching significant DEGs (**Supplementary Table S4**).

Among the 322 significant DEGS, 101 (31.4%) did not have significant matches in the GO, KEGG, or TF databases (**Supplementary Table S5**). However, 62 of the 101 had significant matches in at least one of the NR, NT, or Uniport databases. There were no significant matches across the six databases for the remaining 39 DEGs.

### 3D protein structure prediction

**Figure 11:**
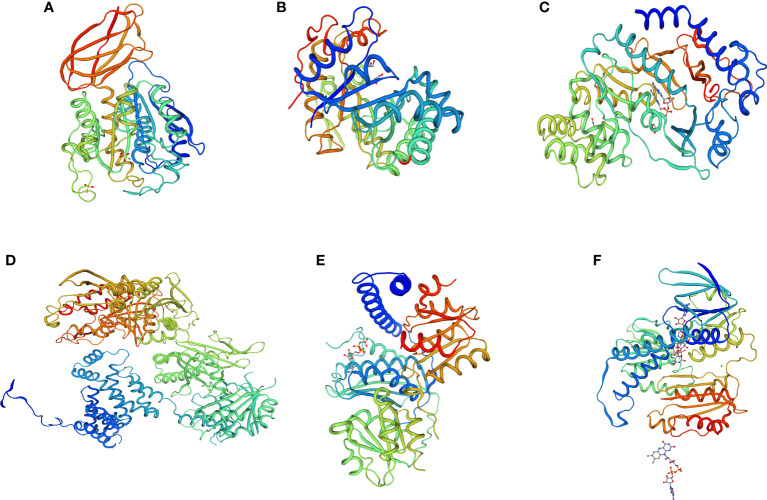
3D protein structure prediction of three downregulated unigenes **(A)** DN399333_c0_g2_i4 (GH3-1 auxin conjugating enzyme), **(B)** DN37014_c0_g1_i1 (pollen allergen CJP38), and **(C)** DN42426_c0_g1_i16 (carboxypeptidase GP 180 residues 503-882), as well as three upregulated unigenes **(D)** DN43311_c0_g5_i10 (acetyl-CoA carboxylase), **(E)** DN45321_c1_g3_i8 (hybrid kinase), and **(F)** DN42426_c0_g1_i16 (dihydrolipoyl dehydrogenase) obtained from the six finger millet genotypes (AT1, AT2, AT3, AS1, AS2, and AS3).

To determine the protein interactions of the upregulated and downregulated unigenes, 3D protein structures were predicted using SWISS-MODEL. The predicted 3D structures of three downregulated unigenes (DN399333_c0_g2_i4, DN37014_c0_g1_i1, and DN42426_c0_g1_i16) showed GH3-1 auxin conjugating enzyme, pollen allergen CJP38, and carboxypeptidase GP 180 residues 503-882 at 68%, 52% and 58% similarity levels, respectively (**Figures 11A–C**). Similarly, the 3D protein structures predicted from three upregulated unigenes (DN43311_c0_g5_i10, DN45321_c1_g3_i8, and DN42426_c0_g1_i16) showed acetyl-CoA carboxylase, hybrid kinase, and dihydrolipoyl dehydrogenase at 62%, 55%, and 64% of similarity levels, respectively (**Figures 11D–F**).

### The distribution of SSR in the unigenes

The unigenes assembled in this study were scanned for both perfect and compound SSRs using MIcroSAtellite (MISA) software. SSRs that contain two or more SSRs of the same or different types separated by up to 100 nucleotides were referred to as compound SSRs. Of the total 198,546 scanned unigenes, 30,561 unigenes (15.4%) contain SSRs of which 6,147 had more than one SSR. In total, 38,090 SSRs were identified across the 30,561 unigenes, of which 35,897 and 2,193 were perfect SSRs and compound SSRs, respectively. Of the 35,897 perfect SSRs, 21,150 (58.9%), 5,694 (15.9%), 8,681 (24.2%), 264 (0.7%), 82 (0.2%), and 26 (0.1%) were mono, di, tri, tetra, penta, and hexa-nucleotide repeat SSRs, respectively (**Table 4**).

**Table 4:** Summary of perfect SSRs identified within the unigenes assembled in this studies: The number of mono, di, tri, tetra, penta, and hexa-nucleotide repeat SSRs for different number of repeats and their corresponding percentage within each SSR type and overall.

SSR type	Repeat motif	Number of repeats											
		5	6	7	8	9	10	11	12	≥13	Total	Freq. (%)^a^	Freq. (%)^b^
Mono-nucleotide repeats	A/T	—	—	—	—	—	9334	3879	1865	4695	19773	93.49	55.08
	G/C	—	—	—	—	—	142	133	139	963	1377	6.51	3.83
Di-nucleotide repeats	AC/GT	—	147	80	60	42	16	9	23	25	402	7.06	1.12
	AG/CT	—	475	304	273	186	131	201	168	279	2017	35.42	5.62
	AT/AT	—	149	61	30	26	18	30	7	58	379	6.66	1.06
	CA/TG	—	183	89	47	32	13	11	9	29	413	7.25	1.15
	CG/CG	—	65	23	6	—	—	—	—	—	94	1.65	0.26
	GA/TC	—	517	324	249	200	116	139	125	231	1901	33.39	5.29
	TA/TA	—	149	44	43	27	20	14	8	53	358	6.29	1.00
	GC/GC	—	101	25	3	1	—	—	—	—	130	2.28	0.36
Tri-nucleotide repeats	CGC/GCG	903	320	95	55	2	—	—	—	—	1375	15.84	3.83
	CCG/CGG	654	230	103	19	4	—	—	—	—	1010	11.63	2.81
	GCC/GGC	650	195	59	44	2	—	—	—	—	950	10.94	2.65
	Others	3255	1138	528	309	31	24	21	14	26	5346	61.58	14.89
Tetra-nucleotide repeats	AGGA/TCCT	16	—	—	—	—	—	—	—	—	16	6.06	0.04
	CCTC/GAGG	11	—	4	—	—	—	—	—	—	15	5.68	0.04
	Others	155	45	13	12	3	1	—	3	1	233	88.26	0.65
Penta-nucleotide repeats	all	70	9	—	3	—	—	—	—	—	82	—	0.23
Hexa-nucleotide repeats	all	16	9	1	—	—	—	—	—	—	26	—	0.07
Total		5730	3732	1753	1153	556	9815	4437	2361	6360	35897		100
Frequency (%)		16.0	10.4	4.9	3.2	1.5	27.3	12.4	6.6	17.7	100		

Freq. (%) ^a^ = percentage among the corresponding SSR type; Freq. (%) ^b^ = percentage among all perfect SSRs.

A/T and G/C repeat SSRs accounted for 93.5% and 6.5% of the mononucleotide repeat SSRs, respectively. Among the di-nucleotide repeat SSRs, the most frequent repeats were AG/CT (2017, 35.4%), and GA/TC (1901, 33.4%), whereas CG/CG (94, 1.6%) and GC/GC (130, 2.3%) were rare. Among tri-nucleotide repeat SSRs, the top three most common motifs were CGC/GCG (1375, 15.8%), CCG/CGG (1010, 11.6%) and GCC/GGC (950, 10.9%) while all others accounted for 61.6% (**Table 4**). Tetra-nucleotide repeat SSRs were diverse, and the top two most common motifs were AGGA/TCCT and CCTC/GAGG accounting for only 6.1% and 5.9% of the tetra-nucleotide repeat SSRs, respectively (**Table 4**).

In the case of the number of repeats of each SSR type, their frequency generally decreased as the number of repeats increased. For instance, the number of mononucleotide repeats with a repeat of 10 was 9476, whereas those with a repeat of 11 were 4012, an over twofold decrease. The number of dinucleotide repeat SSRs with repeats of six and seven was 1786 and 950, respectively, which is a decrease of almost twofold. Trinucleotide repeat SSRs with a repeat of five (5462) were almost three times more frequent than those with a repeat of six (1883, **Table 4**).

Following the exclusion of mononucleotide repeat SSRs, 14,747 SSRs comprising di, tri, tetra, penta, and hexa-nucleotide repeat SSRs were further analyzed in order to identify single-copy SSRs for their various uses in the future. Following this, primers were designed for the identified single-copy SSRs using the Primer3 program. Overall, primer pairs were successfully designed for 3553 single-copy SSRs in 3275 unigenes (**Supplementary Table 6**). Among the 3553 SSRs, 1131 (31.8%), 2339 (65.8%), 59 (1.7%), 20 (0.6%), and 4 (0.1%) are di, tri, tetra, penta, and hexa-nucleotide repeat SSRs.

### SNP, cluster and principal component analyses of the genotypes

The SNP calling for each sample resulted in SNPs that ranged from 329,274 in AT3 to 351333 in AT2. Merging and further filtering of these SNPs resulted in a total of 401,661 SNPs that fulfilled all criteria set to select high-quality SNPs. Out of the 401,661 SNPs, 119,073 were common to all genotypes (**Supplementary Table 7**). Among the 119,073 SNPs, the number of bi-allelic and tri-allelic SNPs was 118,959 (99.9%) and 114 (0.1%), respectively. The polymorphism information content (PIC) of the bi-allelic SNPs ranged from 0.141 to 0.375 while that of tri-allelic SNPs ranged from 0.272 to 0.579. The PIC of 92.9% bi-allelic SNPs and 97.4 tri-allelic SNPs was above 0.30 (**Supplementary Table 7**).

**Figure 12:**
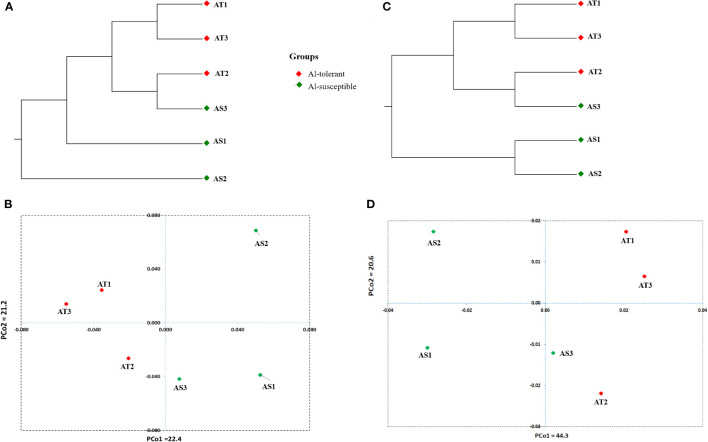
Significantly differentially expressed genes-based UPGMA cluster **(A)** and PCA scatter plot **(B)** depicting the relationship between the finger millet genotypes. Randomly selected 8000 SNPs based UPGMA cluster **(C)** and PCA scatter plot **(D)** depicting the relationship between the finger millet genotypes.

UPGMA cluster analysis and principal coordinate analysis were carried out to determine the genetic relationship between the six genotypes based on 8000 randomly selected bi-allelic SNP markers as well as 417 SNP markers in 113 significant DEGs (**Figures 12A–D**). The cluster and principal coordinate analyses of both data sets revealed that higher genetic variation exists among the Al-susceptible genotypes than among the Al-tolerant genotypes. Furthermore, the analyses showed that the Al-susceptible genotype AS3 is more closely related to the Al-tolerant genotypes than to the other Al-susceptible genotypes. In the PCoA conducted based on the first SNP set (8000 SNPs), the first two principal coordinates explained 43.6% of the total genetic variation between the genotypes, while in the PCoA based on the second SNP set (417 SNPs), 64.9% of the total genetic variation was explained. Both PCoA scatter plots (**Figures 12B, D**) revealed a close genetic relationship between AT1 and AT3, as well as between AT2 and AS3.

## Discussion

Next-generation sequencing (NGS), particularly RNA-seq is a highly effective tool for generating high quality transcriptome sequence reads in several crops such as *Eleusine coracana* (Parvathi et al., 2019), oat (*Avena sativa*; (Kim et al., 2022), sugar cane (*Saccharum officinarum*; (Rosa-Santos et al., 2020) and Japanese carpet shell (*Ruditapes philippinarum*; (Nie et al., 2017). However, no transcriptome-based research has been published on finger millet in association with Al-tolerance. High-quality transcriptome data for Al-tolerant and Al-susceptible finger millet genotypes was generated in this study through RNA-seq to explore differentially expressed genes and identify pathways playing important roles in stress tolerance. The unigenes, SNPs and SSRs generated in this study can serve as permanent genomic resources and tools for various applications in finger millet. It is highly desirable to use the differentially expressed genes (significant DEGs) and SNPs contained in these genes in order to develop efficient sets of markers for marker-aided selection for Al-tolerance in finger millet for the development of novel cultivars for cultivation in acidic soils. These markers could also serve as valuable components of a marker set for genomic selection.

### Annotation of the unigenes

To determine the functions of the identified genes, annotation was performed using the publically available DNA and protein databases. Most of the unigenes (56.5%) had statistically significant hits at least in one of the databases. Nevertheless, a large proportion (43.5%) of the unigenes did not have significant hits. Possible reasons includes lack of sequenced databases for finger millet, short sequence length of some of the unigenes, the presence of non-coding sequences or sequences of untranslated regions, and a high sequence variation that results in an E-value above the cutoff. This is in line with the findings of Desai et al. (2021); Zhang et al. (2021), and (Kim et al., 2022), who found a large proportion of gene sequences that lacked significant sequence similarity hits in various databases.

Among the finger millet unigenes identified in the present study that had significant hits in NR database, 23.7% of these hits were for foxtail millet protein sequences, whereas only 0.2% were for finger millet protein sequences. The high proportion of hits against foxtail millet is expected given that foxtail millet is one of the closest species to finger millet among the plant species that are well represented in the database. In their inter-genome collinearity analysis, Hittalmani et al. (2017) showed that 98% of the finger millet genome is collinear with that of foxtail millet. The fact that only 0.2% of the unigenes had significant hits against finger millet sequences shows that there are few finger millet protein sequences in the database, as is the case for genomic resources of finger millet in other databases. To facilitate comparative and translational genomics of finger millet and, ultimately, genomics-led breeding of the crop, more needs to be done to enrich databases with finger millet omics resources.

Gene ontology functional classification may help us understand the distribution of gene functions, and to predict the role of each unigene. The GO analysis performed in this study classified the genes that had significant hits into 19, 13, and 4 functional subcategories of BP, MF, and CC gene ontology classes. The BP class was dominated by cellular process, metabolic process, biological adhesion, and response to stimulus. MF was dominated by catalytic activity and binding, and CC was dominated by cellular anatomical entity and intracellular entity. The results indicate that the identified genes are engaged in diverse functional, regulatory, and structural activities. Previous studies have shown that cellular processes, metabolic processes, binding of molecules, response to stimuli, as well as metabolism of organic anions are highly correlated with stress tolerance and disease resistance (Hittalmani et al., 2017; Li et al., 2017).

The unigenes were also annotated separately in the KEGG databases to determine their putative functions based on their sequence homology with annotated genes. Among the 89,253 unigenes with significant BLAST hits (E-value cutoff = 1e^-5^) in the KEGG database, 36,696 (41.1%) unigenes were annotated under 201 pathways. These were classified in to cellular processes, environmental information processing, genetic information processing, metabolism, and organismal systems. Most of the unigenes were annotated with the metabolism pathway. The majority of the unigenes predicted were involved in either signal transduction, carbohydrate metabolism, translation, folding, transport and catabolism, lipid metabolism, or sorting and degradation pathways. Being active of these pathway agreed with previous study on Al-stress in *Stylosanthes guianensis* (Jiang et al., 2018).

### Differentially expressed genes

In order to gain deep insights into the mechanism of Al-tolerance in finger millet during their exposure to Al-toxicity, this study was undertaken with the primary objective of identifying differentially expressed genes involved in aluminum tolerance mechanisms. In the present study, Al-tolerance based transcriptomic analysis generated 495 differentially expressed genes, of which 322 genes were significantly differentially expressed between Al-tolerant and Al-susceptible finger millet genotypes. These are a large number of genes although they are fewer than 6165 Al-stress responsive DEGs identified in sugarcane (Rosa-Santos et al., 2020), and 1790 drought responsive DEGs identified in finger millet (Parvathi et al., 2019) using RNA-seq. Due to the use of leaf tissue instead of root tissue for the transcriptome analyses, there may have been fewer significant DEGs identified in this study, indicating that leaves are less responsive to the effects of Al.

As a major Al tolerance mechanism plants secrete aluminum-chelating organic acids malate, oxalate and citrate into the rhizosphere *via* Al-activated malate transporters (ALMT) and multidrug and toxin extrusion transporters (MATE), respectively (Hoekenga et al., 2006; Magalhaes et al., 2007; Liang et al., 2013), thereby detoxifying Al ions and increasing availability of minerals to plants (Kochian et al., 2015). Zinc-finger transcription factors, such as Sensitive to Proton Rhizotoxicity 1 (STOP1) have been reported to regulate this Al resistance mechanism (Fang et al., 2020). In the present study, both ALMT and MATE homologs were either absent or expressed at extremely low levels in the leaves of both Al-tolerant and Al-sensitive genotypes. Meanwhile, STOP1 homologs were highly expressed in both groups. Hence, it would be useful to conduct further transcriptome-based research in order to determine the pattern of expression of these genes under Al stress conditions in different plant organs, including roots and leaves.

In this study, differential expression analysis of RNA-seq data revealed a large number of upregulated and downregulated genes in the Al-tolerant finger millet genotypes. Some genes were upregulated in all Al-tolerant genotypes and downregulated in all Al-susceptible genotypes, and vice versa. GO enrichment analysis revealed important GO calsses such as protein processing (GO:0016485), metal ion transport (GO:00300001), defense response to bacteria (GO:0042742), lipid metabolism (GO:0006629), regulation of meristem development (GO:0048509), and seedling development (GO:0090351).

The unigene DN38351_c0_g1_i1 (GO:0048509) shared a sequence similarity of over 83% with the carboxypeptidase SOL1 gene found in a variety of Poaceae species, including *Panicum halli*, *Setaria italica*, *Sorghum bicolor*, *Oryza sativa*, *Lolium rigidum*, *Zea mays*, and *Triticum aestivum*. Therefore, the unigene shared sequence homolog with carboxypeptidase SOL1 gene. As a result of the gene ontology annotation, the gene has been identified as being involved in metallocarboxypeptidase activity (GO:0004181) as well as the regulation of root meristem growth (GO:0048509). In this study, this gene is downregulated in all Al-tolerant genotypes (log2FC = -7.6). The SOL1 gene encodes a putative zinc carboxypeptidase (Casamitjana-Martınez et al., 2003; Schuldt, 2003). Casamitjana-Martınez et al. (2003) reported that SOL1 functions as a suppressor of the CLE19 gene, which plays an active role in root meristem maintenance. If the expression pattern of this gene is similar in the roots and leaves of finger millet, it would be interesting to examine how the downregulation of this gene contributes to root meristem maintenance.

The unigene DN40309_c0_g2_i3 (GO:0019829) shared sequence similarity of above 82% with cadmium/zinc-transporting ATPase HMA3 (heavy metal associated) gene of various grass species, including *Setaria italica*, *Sorghum bicolor*, *Oryza sativa*, and *Zea mays*. HMA is a member of the ATPase protein family involved in metal ion transport in plants (Liu et al., 2017; Haque et al., 2022). In this study, the gene is upregulated in all Al-tolerant genotypes (log2FC = 7.1), and the GO annotations indicate that it is involved in cation-transporting ATPase activity (GO:0019829) and metal ion transport (GO:0030001). Hence, the gene represented by this unigene is a finger millet homologue of HMA3 gene. The fact that this gene is upregulated in Al-tolerant genotypes indicates that it may be involved in Al-ion exclusion in addition to cadmium and zinc to exclusion zones in order to minimize Al-toxicity and promote Al-tolerance. Further research will shed light on whether the gene is an orthologue of HMA3 or a paralog that has evolved to function in the transport of Al-ion.

The unigene DN40527_c0_g1_i4 (GO:0042742) shared a sequence identity of over 93% with the plastid-lipid-associated protein 6 (PAP) gene found in different crops. This unigene shared homolog with plastid-lipid-associated protein 6 (PAP) gene in various crops (Leitner-Dagan et al., 2006). In this study, the unigene has been being downregulated (Log2FC = -3.3) and is annotated as being involved in the defense response to bacterial activity (GO:0042742). The PAP gene appears to be among a highly conserved group of genes and can be upregulated by various biotic and abiotic stresses factors including droughts. The role of PAP is to sequestrate carotenoids and hydrophobic molecules as well as protect crops from various stress factors (Leitner-Dagan et al., 2006). One of the Al-tolerance mechanisms in plants is that after Al-enters plant cells it will be sequestrated into different cellular compartments such as vacuoles (Kochian et al., 2005).

A unigene DN44795_c0_g1_i3 (GO:0006629) shared 80% sequence similarity with the lipase like Phytoalexin deficient4 (PAD4) gene found in *Arabidopsis thaliana, Panicum hallii, Panicum virgatum, and Setaria italic*. The lipase-like PAD4 gene increase immunity by stimulating the production of the defense hormone salicylic acid (SA) and anti-microbial molecules, which limit pathogen growth (Feys et al., 2005). In this study, the unigene was upregulated (Log2FC = 1.4) and annotated as being involved in lipid metabolism. The expression of lipid metabolism genes supported previous observations that Al can induce the peroxidation of lipids and the production of reactive oxygen species (ROS), and that plant roots are able to increase the expression of antioxidant genes to tolerate Al-stress (Zhu et al., 2015).

Among the top hits for DN43070_c0_g1_i4 (upregulated = Log2FC = 1.4) in the NT and NR databases are putative 1-phosphatidylinositol-3-phosphate 5-kinase FAB1Cs from Poaceae species such as maize and sorghum. The GO enrichment analysis showed that this gene is enriched for phosphatidylinositol phosphate kinase activity (GO:0016307). Similarly, KEGG pathway analysis revealed it is involved in the Phosphatidylinositol signaling system (ko04070) and therefore plays a crucial role in cell physiology through its involvement in signal transduction pathways. The inhibition of phosphatidylinositol phosphate kinase activity by aluminum has been demonstrated in coffee (Martínez-Estévez et al., 2003), and in a recent study in Arabidopsis (Sadhukhan et al., 2021).

The plant hormone signaling system (ko04075) comprised two unigenes (DN39933_c0_g2_i4 and DN43311_c0_g5_i10) and are considered significant players in mediating plant stress response. Moreover, activation of metabolic pathways such as glyoxylate and dicarboxylate (ko00630) as well as starch and sucrose (ko00500) metabolisms, indicating that the plants might use those specific compounds to survive or promote its growth. Activation of glyoxylate and dicarboxylate as well as starch and sucrose were also described in cotton plants (Akbar et al., 2022). Thus, this gene may play a crucial role in Al-tolerance in finger millet.

Transcription factors, such as activators, inhibitors, or both, which regulate the expression of genes have a vital role in various aspects of plant growth and development (Hu et al., 2020). Most of the time, the regulation of TFs differs depending on various factors, such as the type and intensity of stresses as well as the plant species and organs involved. The functional annotations of the DEGs identified in this study showed that a most of them are transcription factor genes, including those encoding AP2, bZIP, MYB, NAC, and WRKY, which were reported to be associated with biotic and abiotic stress tolerance in various crops (Zhu et al., 2013; Huang et al., 2015). This study showed that both upregulated and downregulated genes may belong to the same TF protein family, indicating that TF genes of the same protein family may play contrasting regulatory roles.

Among the DEGs, DN42426_c0_g1_i16 had significant hits in the plant TFDB which corresponds to the AP2 TF of Poaceae species. This gene is downregulated in Al-tolerant genotypes (Log2FC = -7.3) compared to Al-susceptible genotypes. Different transcriptome-based studies have demonstrated that AP2 family proteins play a role in Al-tolerance in plants. Based on their root transcriptome studies, Liu et al. (2021) and Zhao et al. (2020) found an increase in the expression of genes encoding AP2 family proteins in Al-tolerant genotypes compared to Al-susceptible genotypes. Apparently, the DEG identified in this study is not expressed in the leaves of Al-tolerant genotypes, and thus further investigation to determine its expression in the roots is highly desirable.

The DN44845_c0_g1_i2, DN43468_c1_g2_i9, DN38637_c1_g1_i2, and DN43333_c0_g4_i1 unigenes were significantly upregulated in the Al-tolerant genotypes with Log2FC of 6.8, 2.6, 1.9, and 1.13, respectively. DN44845_c0_g1_i2, DN43468_c1_g2_i9, DN38637_c1_g1_i2, and DN43333_c0_g4_i1 unigenes shared sequence similarity with TFs in *Gossypium hirsutum* (Gh_D05G0105), *Trifolium pretense* (TP57577_TGAC_v2_mRNA6397), *Zoysia japonicum* (Zjn_sc00045.1.g01150.1.am.mk) and *Fragaria vesca* (mrna13716.1-v1.0-hybrid), respectively. The upregulation of bZIP genes in this study agreed with previously pulished findings under abiotic stress conditions has been documented (Banerjee and Roychoudhury, 2017). Yang et al. (2011) showed that upregulation of bZIP reduced the uptake and accumulation of aluminum in the roots. Generally, the upregulation of bZIP TFs enhances the upregulation of stress responsive genes *via* abscisic acid (ABA).

DN28660_c0_g1_i3, DN28807_c0_g1_i1, and DN41816_c0_g2_i6) were among the downregulated unigenes with Log2FC value of -2.4, -4.7, -6.5, and -7.1, respectively. The top hit for DN28660_c0_g1_i3 unigene in the plant TFDB is the transcription factor gene of WRKY of *Triticum Urartu* (TRIUR3_28246-P1). DN28807_c0_g1_i1 and DN41816_c0_g2_i6 shared sequence similarity with WRKY TF in *Phyllostachys heterocycle* (PH01003485G0070) and *Ananas comosus* (Aco009877.1), respectively. On the other hand, DN40895_c2_g1_i15 and DN40895_c2_g1_i13 were upregulated unigenes with Log2FC values of 7.06 and 9.05 shared sequence similarity with WRKY TF. The involvement of WRKY TFs in conferring Al tolerance to plants has been reported in different studies. In *Arabidopsis*, WRKY7 has been shown to confer Al tolerance by regulating the expression of genes involved in the modification of cell walls, implicated in a number of developmental processes as well as a defense response against biotic and abiotic stresses (Rushton et al., 2010; Zhang et al., 2011; Li et al., 2020).

Among the significant DEGs, the predicted 3D protein structure of the downregulated unigene DN39933_c0_g2_i4 shared 68% sequence similar with that of GH3-1 auxin conjugate enzyme. Auxin is mainly produced at the tip of the mature leaf through IPyA pathway in most species and then channeled through polar auxin transport (PAT) into root to produce adventitious root and establish new root to replace damaged roots (Cano et al., 2018).

On the other hand, the upregulated DN42426_c0_g1_i16 unigene was 64% similar with dihydrolipolyl dehydrogenase multienzyme complex. Dihydrolipolyl dehydrogenase is required to maintain the metabolic fluxes through the TCA cycle, photorespiration and branched side chain amino acid degradation (Timm et al., 2015). In addition, 3D protein structure predicted another upregulated unigene (DN43311_c0_g5_i10) that shared 62% similarity with acetyl-CoA carboxylase. Acetyl-CoA carboxylase has an important role in the control of plant lipid metabolism (Harwood, 1988) which might reduce the rate of lipid peroxidation due to Al stress.

Other significant DEGs encoding TFs that have been implicated in aluminum or other abiotic stress responses in previous studies include those encoding ERF in *Arabidopsis thaliana* (Li et al., 2022), NAC in *Solanum lycopersicum* (Jin et al., 2020), and MYB in *Zea mays* (Chen et al., 2018). Overall, most of the TFs obtained in this study were also reported in tea plant, rice and Arabidopsis in response to Al-toxicity (Li et al., 2017; Li et al., 2018) and in finger millet in response to drought and for nutraceutical characteristics (Hittalmani et al., 2017). Further studies investigating the role of these and other significant DEGs identified in the present study would lead to their applications in genomics-led breeding for Al tolerance of finger millet.

### SSR and SNP markers

Transcriptome-derived SSRs, such as EST-SSRs have been developed for various crop species such as noug, sugar beet, and wheat (Fugate et al., 2014; Yang et al., 2016; Tsehay et al., 2020). The present study showed the presence of more trinucleotide repeats than dinucleotide repeats in the finger millet transcriptome. The result is in agreement with previous reports on monocots such as finger millet, rice, Siam tulip, *Curcuma alismatifolia*, and common reed *Phragmites karky* (Lawson and Zhang, 2006; Kumar et al., 2015; Taheri et al., 2019; Nayak et al., 2020). Arabidopsis genes were also found to contain more trinucleotide repeat SSRs than dinucleotide repeat SSRs (Morgante et al., 2002). The most plausible explanation for a higher frequency of trinucleotide SSRs than dinucleotide SSRs within transcriptome sequences is the fact that they do not cause frameshift of coding sequences. AG/CT and GA/TC dinucleotide repeats and CGC/GCG and CCG/CGG trinucleotide repeats were the most abundant repeat motifs revealed in this study, which were also common in other monocot crops, such as little millet (Desai et al., 2021), bread wheat (Yang et al., 2016) and radish (Zhai et al., 2014). The abundance of CCG/CGG motifs in finger millet is consistent with previous findings in monocots (rice, maize, and wheat) (Morgante et al., 2002). In contrast, CCG/CGG motifs are rare in dicots (Arabidopsis, soybean, and noug) (Morgante et al., 2002; Gebeyehu et al., 2022). Tetra, penta, and hexanucleotide SSRs represent only 1% of all SSRs in the present study, indicating that they are relatively rare in finger millet transcriptomes. In a similar study conducted on noug, these SSRs accounted for 2.7% of the total SSRs identified (Gebeyehu et al., 2022). In the case of the number of repeats of each SSR type, their frequency generally decreased as the number of repeats increased, which is consistent with various previous studies.

The G+C content in finger millet unigenes (46.4%) is higher than the G+C content of the SSRs derived from these unigenes (34.1%). A similar pattern was reported in *Populus* (Li and Yin, 2007) and noug (Gebeyehu et al., 2022). The G+C content of the mononucleotide repeat SSRs of finger millet (8.8%) revealed in this study was higher than that of noug (3.9%; Gebeyehu et al., 2022). Similarly, the G+C contents of dinucleotide (45.0%) and trinucleotide repeat SSRs (70.5%) of finger millet (a monocot) were considerably higher than that of noug (a dicot). The higher G+C content of trinucleotide SSRs is a consequence of their higher frequency in GC-rich gene sequences. It is also interesting to note that the G+C content of the finger millet SSRs (34.1%) was higher than the 22.2% G+C content found in noug in a similar study (Gebeyehu et al., 2022), which is in line with the overall higher G+C content of monocot genomes than dicot genomes.

In this study, highly informative SNP markers were generated based on the transcriptome data of the finger millet genotypes. The PIC of 92.9% bi-allelic SNPs and 97.4 tri-allelic SNPs was above 0.30 indicating that the vast majority of the markers are highly informative. As finger millet is an orphan crop and has not been well studied at the genomics level, this data is highly suitable for use in various applications, including population genetic analyses for conservation and breeding, marker-trait association studies as well as genomic predictions for desirable traits. Marker-trait association studies using these markers targeting desirable traits will eventually lead to the identification of a core set of markers for the development of an SNP-chip that can be used to facilitate genomics-led breeding of finger millet. As revealed through cluster and principal component analyses conducted based on randomly selected SNPs as well as SNPs within DEGs, there was high genetic variation among the finger millet genotypes, particularly among the Al-susceptible genotypes. The result is in line with previous SSR and SNP marker based studies on Finger millet (Brhane et al., 2021; Brhane et al., 2022). Despite the small number of samples, the fact that more genetic variation was detected in Al-susceptible genotypes than in Al-tolerant genotypes suggests that there is limited diversity in Al-tolerant finger millet.

Among the 322 significant DEGs, six of them showed genotypic differences between the Al-tolerant and Al-susceptible genotypes (**Supplementary Table S7**) at specific SNP loci. It is interesting to note that in all cases, one of the two groups was heterozygous while the other group was homozygous for one of the two alleles, indicating the absence of homozygous genotypes for the other allele. These SNP loci are of interest to investigate in a larger number of Al-tolerant and Al-susceptible genotypes in order to determine whether they can be used as expression markers (eMarkers) to assist in the selection of Al-tolerant genotypes.

## Conclusion

By providing high-quality transcriptome sequences, SNPs and SSR markers as well as revealing differentially expressed genes between Al-tolerant and Al-susceptible finger millet genotypes, RNA-seq has proven to be an effective NGS method. The study identified high-quality genomic resources consisting of 198,546 unigenes, 3,553 single-copy SSR markers, and 119,073 SNP markers. About 56% of the unigenes had significant hits in one or more genomic databases. The fact that only 0.26% of the unigenes had significant hits against finger millet sequences in the NR database indicates the scarcity of finger millet genomic resources. In this regard, the data generated in this study make a substantial contribution to enriching the public genomic databases for finger millet, which facilitates future research on this crop. In total, 322 significant DEGs were identified in this study, of which 131 were upregulated and 191 were downregulated in genotypes that are Al-tolerant. The major activities and processes associated with the DEGs include catalytic activity, binding, transport activity, response to stimuli, and metabolic processes such as glyoxylate and dicarboxylate metabolism. Among the DEGs identified, a significant proportion encode transcription factors, such as AP2, bHLH, bZIP, C2H2, C3H, ERF, GATA, MYB, NAC, and WRKY. Among the significant DEGs are those that have been previously reported to be associated with Al tolerance or other abiotic stress responses in different crops. These include homologs of carboxypeptidase SOL1, HMA3, 1-phosphatidylinositol-3-phosphate 5-kinase FAB1C, as well as AP2, bZIP, C3H, and WRKY TF genes. Further investigations into these and other significant DEGs will provide detail insights; thereby facilitate genomic-led breeding for Al tolerance in finger millet. The distribution of different SSR types in finger millet was similar to that of other monocot crops, with trinucleotide repeats being more prevalent. A vast majority of the SNPs had PIC values above 0.3, indicating that they are highly informative. Among their many applications, these markers are essential genomic resources for the transition from traditional plant breeding to genomics-driven breeding methods for the development of new finger millet cultivars, including those that are Al tolerant.

## Data availability statement

The datasets presented in this study can be found in online repositories. The names of the repository/repositories and accession number(s) can be found in the article/**Supplementary Material**.

## Author contributions

Conceptualization and methodology, HB, MG, TH, KT, CH and RO. Data collection, HB, MG, CH and RV. Data analysis, HB under guidance of MG, CH and KBA. Original draft preparation, HB. Review and editing, HB, TH, KT, CH, RO, KBA, RV and MG. Project funding acquisition and administration, TH, KT, RO, CH and MG. All authors contributed to the article and approved the submitted version.

## Supplementary material

Supplementary Figure 1 Histogram showing **(A)** the top 20 most frequently hit transcription factors, and **(B)** the top 20 most frequently hit plant species.

Supplementary Table 1 The List of 495 (sheet 1) differentially expressed unigenes and 322 significant DEGs (sheet 2) between Al-tolerant and Al-susceptible groups and their annotations in different databases with adjusted p-value< 0.1; Log_2_FC< -0.5 or > 05 and adjusted p-value< 0.05; Log_2_FC< -1 or > 1, respectively.

Supplementary Table 2 List of significantly differentially expressed unigenes between Al-tolerant and Al-susceptible groups and their annotations in GO database.

Supplementary Table 3 The List of 60 significantly differentially expressed unigenes between Al-tolerant and Al-susceptible groups and their annotations in KEGG Pathway database.

Supplementary Table 4 The list of 137 significantly differentially expressed unigenes between Al-tolerant and Al-susceptible groups with annotation in the Transcription factor (TF) database. Unigenes referring to the same TF family proteins are consecutive and highlighted with the same color.

Supplementary Table 5 The list of 62 significantly differentially expressed unigenes between Al-tolerant and Al-susceptible groups that were not annotated in GO, KEGG and TF, but had signficant sequence matches at least in one of NR, NT and Uniport databases.

Supplementary Table 6 List of 3553 single-copy SSRs in 3275 unigenes, and their corresponding size, primer-pairs and reference sequences. The number of di, tri, tetra, penta and hexanucleotide repeat SSRs are 1131, 2339, 59, 20, and 4, respectively. Note: Highlighted (conditional formatting) unigenes in consecutive rows are the same.

Supplementary Table 7 List of 417 SNP loci in 113 significantly differentially expressed genes (significant DEGs) among the Al-tolerant and Al-susceptible genotypes and their corresponding reference and alternate alleles, position in the unigene, minor allele frequency (MAF), polymorphism information content (PIC), and unigene sequence, as well as genotypes of the six samples. The genotypes of Al-tolerant and Al-susceptible genotypes differ at the green highlighted loci.
