# Supplementary figures and images for: Finger millet RNA-seq reveals differential gene expression associated with tolerance to aluminum toxicity and provides novel genomic resources
Source: Front Plant Sci. 2022 Dec 9;13:1068383. doi: 10.3389/fpls.2022.1068383 (PMC9780683; doi:10.3389/fpls.2022.1068383)

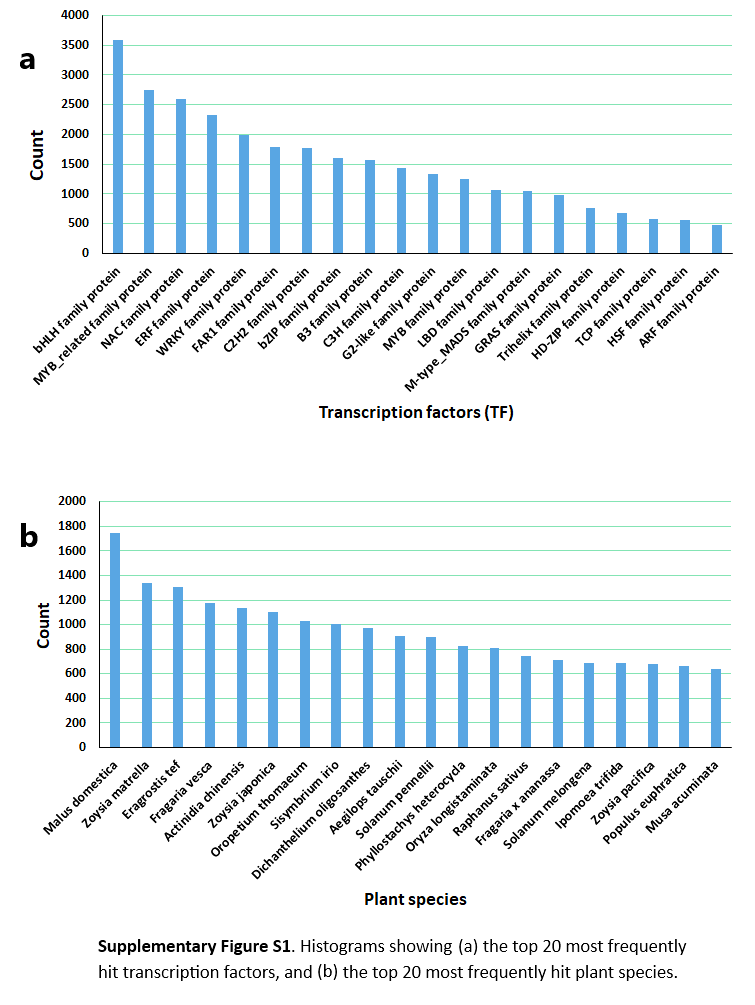

Supplement: Supplementary Figure 1 — Histogram showing (A) the top 20 most frequently hit transcription factors, and (B) the top 20 most frequently hit plant species. [file Image_1.tiff]
